# The role of community health workers in palliative care in a rural subdistrict in South Africa

**DOI:** 10.4102/phcfm.v14i1.3657

**Published:** 2022-11-09

**Authors:** Elza M. van Heerden, Louis S. Jenkins

**Affiliations:** 1 Department of Family and Emergency Medicine, Faculty of Medicine and Health Sciences, Stellenbosch University, Cape Town, South Africa; 2 Department of Family, Community and Emergency Medicine, Faculty of Medicine and Health Sciences, University of Cape Town, Cape Town, South Africa; 3 Department of Family and Emergency Medicine, George Hospital, Western Cape Department of Health, George, South Africa

**Keywords:** palliative care, pain, community health workers, roles, rural

## Abstract

**Background:**

Effective palliative care is an urgent humanitarian need, particularly in less developed countries, including South Africa (SA). People can be palliated within their communities, motivating the integration of palliative care into primary healthcare systems. While community health workers (CHWs) play a vital role in health coverage at the primary care level, literature on their roles in palliation is limited.

**Aim:**

To explore the roles of CHWs in palliative care delivery in a rural subdistrict in SA.

**Setting:**

This study was conducted in the George subdistrict of the Western Cape province, SA.

**Methods:**

A descriptive qualitative study explored the perceptions of a wide range of stakeholders (*n* = 39) of CHWs’ roles in palliative care. Data were collected via semistructured interviews and focus group discussions and analysed thematically.

**Results:**

Patients experienced severe biopsychosocial symptoms and needed home-based palliation. While CHWs identified and referred patients, their main responsibilities were health promotion and disease prevention. Palliation was primarily a registered nurse’s function. Community health workers were conflicted by their limited ability to deliver basic palliative care to patients.

**Conclusion:**

While there is a definite need for community-based palliative care, the optimal structure of such a service and the roles of CHWs therein are uncertain. Future research should explore the home-based palliation needs of patients in similar contexts and the service design best suited to address these needs within the primary healthcare domain.

**Contribution:**

This study illustrates the influence of individual and system-related factors on CHWs’ roles in palliative care. It can inform service design to optimise CHWs’ contribution to palliation within primary health care.

## Background

Palliative care aims to prevent and relieve suffering and is considered ‘an urgent humanitarian need’ for those living with chronic fatal diseases.^1^ The effective management of pain is regarded as an essential component of good palliation,^2^ while the advocacy of palliative care and pain treatment as fundamental human rights underscores its significance.^3^ While good palliation is a universal health issue, it is of specific importance in developing countries, as an estimated 77.5% of worldwide deaths have occurred in less developed regions (including all of Africa) from 2015 to 2020.^4^

The high symptom prevalence and burden amongst patients with palliative care needs in sub-Saharan Africa emphasise the need for effective palliative care. A study conducted in five palliative care facilities (four in South Africa [SA] and one in Uganda) reported that 87.5% of cancer patients and 82.6% of patients with human immunodeficiency virus (HIV) experienced pain.^5,6^ Other prevalent symptoms in both patient groups were lack of energy, drowsiness, sadness and worrying.^5,6^ A more recent study investigating the palliative care needs of people living in an informal settlement in Tshwane, SA, likewise found pain to be the most prevalent symptom, with 89.6% of participants in need of pain relief.^7^ Other common physical symptoms included weakness, fatigue, cough and weight loss.^7^ Psychosocial distress was frequent, with 85.4% of participants requesting emotional support and 50% financial assistance.^7^

In 2017, palliative care delivery in SA was regarded as only being at a preliminary stage of integration into mainstream healthcare services.^8^ In the same year, SA made meaningful progress towards the provision of accessible palliation to especially resource-poor communities with the approval of the National Policy Framework and Strategy on Palliative Care (NPFSPC).^9^ The policy foresees that most patients will receive palliative care within their communities, thus placing palliative care firmly within the domain of primary healthcare. Palliative care is correspondingly included as service responsibility in the SA Department of Health’s 2018 Policy Framework and Strategy for Ward-Based Primary Healthcare Outreach Teams (WBPHCOT), a key component of SA’s primary healthcare re-engineering strategy.^10^

Community health workers (CHWs) have a vital role to fulfil in achieving universal health coverage at the primary care level.^11^ As part of WBPHCOTs, a group of CHWs supported by a registered nurse work as a team to serve the population of a specific geographic area.^12,13^ For home- or bedbound patients, CHWs and home-based carers (HBCs) may well be their main healthcare contact. The NPFSPC fittingly lists the identification and referral of patients with palliative care needs, the control of pain and other symptoms and home nursing as this cadre’s palliative care responsibilities.^9^ Community health workers are primarily involved in patient identification and referral, while HBCs are responsible for caregiving tasks.^9^ The WBPHCOT Policy Framework and Strategy considers CHWs’ core responsibilities to be health promotion, disease prevention and basic therapeutic, rehabilitative and palliative care delivery.^10^ Finally, although considered as national directives, individual provinces have some autonomy in implementing state policies.^14^ ‘Healthcare 2030: The Road to Wellness’ describes the Western Cape province’s long-term provincial health strategy.^15^ It recognises home- and community-based care (HCBC) as a core component of the province’s primary healthcare services.^15^ Prevention and health promotion are the main objectives, with a complementary capacity for curative, rehabilitative and palliative care.^15^

Despite the availability of relevant legislature, little is known about the actual contribution of CHWs to palliative care delivery in SA. A single study on the palliative care roles of CHWs in a rural community in the Limpopo province of SA reported that they can meaningfully contribute to the provision of physical comfort, spiritual support, patient education and medication management.^16^ Two further studies investigating the palliative care learning needs of SA CHWs suggested a similar role capacity.^17,18^ The aim of this study was to explore the roles of CHWs in palliative care in a rural subdistrict in SA.

## Methods

This was a descriptive exploratory qualitative study using semistructured interviews and focus group discussions (FGDs). By exploring the lived experiences and perceptions of a wide range of stakeholders, the aim was to gain a multifaceted and contextual understanding of CHWs’ roles in palliative care delivery.

The study was conducted in the George subdistrict of the Western Cape province, SA. With an estimated population of 218 381, George has the highest gross domestic product per capita in its district, as well as a high level of socio-economic development.^19^ There is marked income inequality, with a projected unemployment rate of 14.3% and an estimated 17.3% of households situated in informal settlements.^19^

In 2018, the George subdistrict became an implementation site for a rural palliative care model.^20^ Community-based services form an integral part of this model, with three nongovernmental organisations (NGOs) employing the CHWs delivering HCBC in the area. While employed by the NGOs, all CHWs are funded by the Western Cape Department of Health (WCDOH). Those appointed before 2016 worked as HBCs and were subsequently redeployed as CHWs (no HBCs were retained). Twenty-eight CHWs are in the service of an NGO with a strong hospice and palliative care background. The local branch of a national NGO specialising in family support employs 12 CHWs, and an NGO initially established to care for HIV, AIDS and tuberculosis patients employs a further 30 CHWs. These organisations are supplemented by three community day centres, 10 fixed and four mobile primary health care clinics, an intermediate care facility as well as one regional, one tuberculosis and one district hospital.^19^ Finally, the primary researcher is a medical doctor with experience in palliative care. Residing in George, she works in the private health sector and is not directly involved with the participating NGOs, their employees or their patients.

Four stakeholder groups, namely CHWs, CHW supervisors, local officials involved in the implementation of CHW policies and patients living with a life-limiting illness and their primary caregivers were included in the study via purposive sampling. To ensure a wide range of perceptions and experiences, CHWs and supervisors employed for longer than 6 months by any of the three relevant NGOs were invited to partake in the study. Patients were recruited through service providers involved in the George rural palliative care project. They had to be using morphine for pain relief but not in obvious distress or moribund. As patients and their caregivers were interviewed in their homes, they had to live in safe and accessible areas.

Data were collected through four FGDs and 10 semistructured interviews during 2021. A FGD was held with the CHWs of each of the three participating NGOs; a follow-up FGD was conducted with one of the participating CHW groups to clarify certain aspects of the information they provided during their initial FGD. Interview guides exploring the perceptions of policy implementers, supervisors and CHWs of the roles of CHWs in palliative care and pain management, as well as the experiences of palliative care patients and their primary caregivers of home-based palliative care and pain management, directed the discussions. Participating patients who had not been visited by CHWs related their perceptions rather than experience of palliative home-based care. This was inevitably because of system factors regarding the changing roles of CHWs. The primary researcher conducted all interviews in either English or Afrikaans, as was determined by the participants’ preference. An interpreter was present to assist in translation to isiXhosa when required. All sessions were audiotaped to generate verbatim accounts of information provided by the interviewees. Relevant demographic data were collected from each participant before their interview.

Interviews were transcribed verbatim and cross-checked for accuracy against the audiotapes by the principal researcher. Guided by the framework method, a process of data familiarisation identified keywords and recurrent issues in the raw data, from which a descriptive coding index was developed. To this end, all transcripts were separately analysed and annotated. Codes were reduced to categories, from which themes and subthemes were developed. Verbatim quotes were used to maintain the voices of the participants and to provide thick descriptions.

### Trustworthiness

To ensure trustworthiness, credibility of the research was achieved by exploring the opinions of a wide range of purposively selected stakeholders. The use of various interviewing techniques, the capturing of data through audio recording and the cross-checking of transcribed data against audiotapes further supported credibility. Transferability was enhanced by the detailed description of the study setting and the participants. Dependability was ensured through iterative data collection until data saturation was reached. Triangulation of the data with published literature and maintaining a clear audit trail from audiotape to transcript to analysis allowed for confirmability. Finally, reflexive practice by the principal researcher and regular discussions with the research supervisors minimised personal biases.

### Ethical considerations

Ethical approval was obtained from the Health Research Ethics Committee of Stellenbosch University (reference number S20/07/155). Permission was also obtained from the Western Cape Department of Health (reference number WC_202103_014) and the three participating NGOs employing the CHWs, as well as informed consent from all participants.

## Results

A total of 39 people participated in the study, most of whom were CHWs (*n* = 23). All the participating patients suffered from advanced cancer. Participants’ demographic details are summarised in Table 1.

**TABLE 1:** Participants’ demographic details.

Participant group	Number	Gender	Age (years)			Employer	Job years		
			Mean	s.d.	Range		Mean	s.d.	Range
CHWs	23	Female: 23	36	11.86	23–63	NGO 1: 8	5	5.53	1–25
	-	-	-	-	-	NGO 2: 7	-	-	-
	-	-	-	-	-	NGO 3: 8	-	-	-
CHW supervisors	5	Female: 5	38	11.77	22–54	NGO 1: 3	3	3.49	1–11
	-	-	-	-	-	NGO 3: 2	-	-	-
Policy implementers	1	Female: 1	54	-	-	WCDOH: 1	5	-	-
Patients living with a life-limiting illness	5	Female: 3	62	15.13	38–81	N/A	N/A	-	-
	-	Male: 2	-	-	-	-	-	-	-
Primary caregivers	5	Female: 4	52	18.56	27–78	N/A	N/A	-	-
	-	Male: 1	-	-	-	-	-	-	-

CHW, community health worker; NGO, non-governmental organisation; WCDOH, Western Cape Department of Health; s.d., standard deviation.

Four major themes and a number of subthemes emerged (see Table 2). Themes related to the community’s palliative care needs, CHWs’ roles in palliative care provision and pain management and the barriers and enablers to these roles.

**TABLE 2:** Major themes and subthemes.

No.	Themes and subthemes
1	There is a need for palliative care in the community Patients and their caregivers lacked information on palliative care Poor socio-economic circumstances intensified communities’ palliative care needs. Seriously ill patients found it hard to attend primary health care clinics. The intermediary care facility does not have the capacity to admit all patients with palliative care needs. Not all patients desired admission.
2	CHWs are uncertain about their roles and responsibilities in palliative care CHWs’ primary function is now disease prevention and health promotion. CHWs who previously worked as HBCs found it challenging to adjust to their new scope of practice. Communities were critical of their new job responsibilities.
3	Barriers to CHWs’ roles in palliative care and pain management CHWs need formal palliative care training. Not all CHWs are suited for or keen to provide palliative care. CHWs were deeply affected by patients’ suffering. Being part of the communities blurred the boundaries between CHWs’ working and private lives.
4	Enablers to CHWs’ roles in palliative care and pain management CHW have an excellent understanding of patients’ everyday circumstances. CHWs who previously worked as HBCs are able to provide patients with information on symptom management and basic care procedures. CHWs have a strong internal motivation and passion for their work.

CHW, community health worker; HBCs, home-based carers.

### There is a need for palliative care in the community

Participants agreed that there was a definite need for palliative care in the community. The demand for this service increased following the 2018 implementation of the local rural palliative care model, with more patients being identified and referred for out-of-hospital palliation:

‘I don’t know years before, but palliative, we started having more referrals from George Hospital more recently. I think 2019.’ (Supervisor, S5)

Advanced disease with a heavy symptom burden, including pain, was prevalent:

‘Even if we are going to the house, she would cry, even if we didn’t go into the room. She was crying like never before. She doesn’t even want us to touch her. She was just lying with her back the whole day and night … She was seriously in pains … Sjoe, I can’t even tell.’ (CHW, C11)

Patients and their primary caregivers generally lacked information on the management of symptoms and treatment-related side effects. Fear of morphine and poor treatment adherence were common occurrences:

‘I was not comfortable with the morphine. I never liked the morphine. I thought that morphine kills you. Affects your organs and kills your organs.’ (Patient, P4)

‘So each and every time when he felt pain, he will take the morphine. Because of what he had heard, the morphine is gonna ease the pain. So he will just take the morphine. And yes, then the pain would ease. But then he didn’t even measure it.’ (CHW, C14)

Poor socio-economic circumstances intensified communities’ palliative care needs. People who received HCBC experienced financial hardship and battled to meet the extra expenses associated with their family member’s advanced disease:

‘She said no, she has no one to ask. If there’s no money, there’s no one to help her.’ (CHW, C5)

‘They will be in that poverty situation waiting for that R1000-something of the pension so that they can live their lives … She’s the only breadwinner because of this pension. Then when she dies, what about the others?’ (CHW, C9)

Patients and their caregivers confirmed this observation:

‘We cannot meet all my mother’s needs. For example, I wanted to make her some jelly. I must be able to make her some custard. Something like – soft foods. I must make her some Weetabix. I must have milk, I must have sugar, all that kind of stuff.’ (Primary caregiver, PC1)

Community health workers reported poor social support structures within families, with cases of extreme neglect and abuse within the community:

‘Sometimes the family just steps away and let the person be. Especially the person that is aggressive, they will just let them be.’ (CHW, C9)

‘When we came there the worms were – his whole body was full of worms. He was just like this – so thin. You could see through; there were holes. He got bedsores.’ (CHW, C8)

Community health workers thought families lacked the necessary knowledge, skills and resources to care for their seriously ill loved ones on their own:

‘So I will say they are not trained … They do not know if they must give food or what. And they do not know how to work with him, because he’s in pain.’ (CHW, C22)

Most patients and their caregivers felt ill-equipped to deal with disease-related symptoms, which increased their psychological distress. Families confirmed their need for continued advice and support on how best to care for their loved ones as their disease progresses:

‘It’s all sort of left on the person who’s battling … But you know, you’re battling with something you don’t know; there is uncertainty. You don’t know what to do, and you don’t want to bother other people. So it’s just an extra stress, that’s all.’ (Primary caregiver, PC5)

‘And that is what one needs. You need someone who will be there for you, someone to whom you can turn.’ (Primary caregiver, PC3)

Regarding alternatives to home-based care, CHWs and caregivers thought that seriously ill patients found it hard to attend primary health care clinics:

‘There are people that can’t come to the clinics. Yesterday morning, the uncle came to me; you can see it’s two old people. He came to collect [*medication at the clinic*] and he said, “She’s bedridden, how am I going to get her here?”’ (CHW, C5)

‘Perhaps you sit and wait for an hour. And I am not saying that my mother is better than other people, but for me, she is the most important. And it is difficult to just go and sit at the clinic with a person in such a condition.’ (Primary caregiver, PC1)

Participants explained that the town’s intermediary care facility, Bethesda Care Centre, did not have the necessary capacity to admit all patients with palliative care needs. Furthermore, not all patients desired admission to a care facility:

‘We refer to Bethesda, but Bethesda cannot keep them too long. So that’s another challenge.’ (Supervisor, S5)

‘Also the fact that she didn’t want to go to Bethesda. The hospital again told me on Monday that if she wants, if we want to take her to Bethesda, then we can … But I don’t feel like doing it, to disrespect my mother’s wishes in such a way.’ (Primary caregiver, PC1)

### Community health workers are uncertain about their roles and responsibilities in palliative care

While thoroughly aware of the growing demand for palliative care, participants experienced their obligations towards service delivery to be in a period of transition, leaving them uncertain about their roles and responsibilities in addressing this need:

‘It was made very clear to us that our focus is not palliative patients. It is more screening and health promotion … With all the palliative care patients being referred now, I am not sure how it is going to work in future if it is not part of our job. How do I say no? Who do I choose?’ (Supervisor, S2)

Community health workers’ primary function being disease prevention and health promotion through community screening, referrals and education presented a major change to the NGOs’ service structures:

‘We are forced to do less thereof [*physical caregiving*] because our work is prevention, promotion and wellness, and that is what we are paid for … It is to push the community to live healthier, to live better and to take care of themselves, rather than for the Department to pay us to do physical care.’ (Supervisor, S3)

The level of CHW involvement in palliative care delivery varied between the participating organisations. In some cases, palliative care home visits were a function of the registered nurse, with concerns raised about the sustainability of this arrangement:

‘When I go to a patient, it is I and Doctor and the social worker, and we manage the whole situation. I do not involve them [*CHWs*] to go with.’ (Supervisor, S3)

‘Sister is one person alone and she can’t handle the whole area. She’s one person, she’s gonna be burnt out.’ (CHW, C3)

The CHWs of other NGOs contributed more to the palliative care needs of patients. Guided by their nursing supervisor, they were involved in the ongoing monitoring of patients and, amongst other tasks, ensuring that patients had sufficient medication supplies:

‘What happens is that I have to go to that house and do the assessment, going with the CHWs that is working in that area. Because what will happen, we will assess the patient and then we work according to the patient’s need. We draw up a care plan.’ (Supervisor, S5)

Community health workers’ current job description did not include any definite palliative care functions. Although they may assist patients with their basic care needs, their scope of practice agreements allotted only 10% of their time to patient care. Their primary goal was to educate patients’ families to care for their loved ones themselves:

‘We are not visiting palliative care patients. We are doing the screening, but we can’t assist the patient, like even what we did in the old days.’ (CHW, C8)

‘And what we are doing mostly, it’s not like the time when there used to be bed washes. We are no longer doing that. We are promoting self-care now. So we educate the family members to assist the patient.’ (Supervisor, S5)

Most CHWs who previously worked as HBCs found it challenging to adjust to their new scope of practice. They were uncertain about their roles and responsibilities and experienced a loss of job-related identity:

‘When I wake up in the morning, I feel that I don’t know where I’m going; I don’t know what I’m going to do. Because on previous times when you wake up, you wake up and then you know that where are you going. What are you going to do … So they take it, they take it, that thing of me. Now I’m – they try to change me to be another person.’ (CHW, C2)

While most CHWs considered educating the next of kin a core part of their responsibilities, they found it difficult to transfer all caregiving tasks to the family and were stressed by their inability to help patients in need of physical care:

‘Then everything changed … Because now we apparently must just show people what to do.’ (CHW, C22)

‘Because we must just talk. We cannot just talk to a patient lying there, in pain and needing a wash.’ (CHW, C3)

When they did provide basic care, the time allowed for this was often too short, and CHWs resorted to helping patients after hours. They also felt that the administrative responsibilities associated with their work took precedence over patient care:

‘Sometimes we do not get to everything, and then we must see to make a plan for the next day. Or if I know it’s a serious problem, I will visit the patient after work.’ (CHW, C22)

‘We are just here now for stats. Because if you have one house with seven people on your daily [*activity sheet*], it’s not enough. So we are running for numbers. We are not running to save people’s lives. We’re not running to help our community.’ (CHW, C5)

According to CHWs, their communities were critical of their new job responsibilities. Screening visits were not always welcomed, and they were still expected to perform basic care procedures. Community health workers were negatively affected by this critique:

‘Our people still expect that we will visit them perhaps two times or three times a week. And they do not understand that our work has changed completely and yes, they still have that expectation. And if those people then say to us, “You can just as well leave it. Why did you come out at all? You don’t mean anything anyway.” Do you know how bad one feels?’ (CHW, C22)

### Barriers to community health workers’ roles in palliative care and pain management

Participants felt that if CHWs needed to fulfil a more definite role in palliative care delivery, then the current absence of formal palliative care training would be a concern. Supervisors agreed that dedicated palliative care training would be of value:

‘Since 2016, our CHW training has focused on asking questions, identifying problems and referring to clinics. This is still the focus – more prevention and information than caring.’ (Policy implementer, O1)

‘I think it would help if they can get training on palliative care. Because sometimes they do go there [*patients’ homes*], but they are not fully equipped of what they must do with that patient.’ (Supervisor, S5)

The possibility that not all CHWs would be suited for or keen to provide palliative care was voiced:

‘I think if one can possibly assign people. Because I do not think everyone would like it.’ (Supervisor, S2)

‘Because some of us, we are not fit for a home-based, ja … I’m not fit. Because as she’s saying, I was crying. I was crying every day we go there. I’m crying.’ (CHW, C12)

Community health workers were deeply affected by patients’ suffering. The fact that this stressor often went unacknowledged increased their burden:

‘So when I leave a patient like that, I go home and I think and I feel the pain. I don’t even worry about my child, because I’m so emotional about this client’s pain.’ (CHW, C5)

‘Because you get attached to them, as they get attached to you. Because we form a bond. But according to our management, we are not allowed to do that. We are not allowed. We must not get involved.’ (CHW, C22)

Being part of the communities they served blurred the boundaries between CHWs’ working and private lives. Given the CHWs’ lay status, healthcare professionals did not always value their opinions. Their unsafe work environments were also not appreciated:

‘The problem is when we are at our houses, the clients still need us. They come to our houses although it’s your time for your children and your husband.’ (CHW, C2)

‘There’s a poverty here, there’s no something to eat. Then you end up, sometimes you say, “You must come to my house, you must send a child, you must come to my house to take something.” A small thing to eat, né. Because you can’t leave an old lady, she’s waiting an old [*age*] grant and the month is long. There’s nothing to eat now.’ (CHW, C16)

‘We know we are not professionals. We know we are not – we don’t have a number to attach to our name. End of the day, we are not professional. But I am a professional when I’m doing my work.’ (CHW, C5)

‘They [*CHW employers*] don’t even notice us. They just say, “It’s your job to walk.” Even if the dog bites you three times. Even if they rob you. They don’t care. They just say it’s your job to walk. That’s all.’ (CHW, C17)

‘We are not looking for sympathy. We are only looking for a little bit of recognition.’ (CHW, C18)

### Enablers to community health workers’ roles in palliative care and pain management

Because CHWs lived in the communities they worked in, they had an excellent understanding of patients’ everyday circumstances. Their perceptions were often more accurate than the information visiting healthcare professionals might have gained:

‘I am the worker of the community. I am the one seeing, living, reliving what they are living. I am telling you, but this is happening like that. Now you go for 1 day. I will tell the family, “Sister is coming on this day”. And that family member will take out that client and make him so good when Sister is coming there. Tomorrow, when Sister is not there, it’s the same thing that I’m seeing, and then that client is not being helped.’ (CHW, C5)

They appreciated patients’ cumulative suffering and were able to identify those with unmet physical, psychosocial and spiritual needs. They also were aware of and utilised community resources when necessary:

‘So you’ll find out it’s not only the cancer that is killing this client. It’s the stress, it’s the place that she lives in, the conditions some clients live under.’ (CHW, C9)

‘Some of them, you see the situation of the house. And then you see, okay, they need a food parcel. They need someone who gonna come and talk. They need a pastor here.’ (CHW, C2)

Community health workers who previously worked as HBCs could provide patients with information on symptom management and basic care procedures. They could recognise uncontrolled pain and misperceptions regarding pain management:

‘It doesn’t help to panic, because the people depend on you. You are here now; you must show them how to make the bed with the patient who cannot move. You must show them how to turn the patient. You must show them how to care for the bedsores. How to manage the pain and diet and everything.’ (CHW, C23)

Community health workers had a strong internal motivation and passion for their work. They considered patient care as their core skill and responsibility:

‘The CHWs do have a passion to work with people. They do not hesitate to help or to assist when it is needed.’ (Supervisor, S4)

‘So there is a something that bring you till here. You are not coming just with walk to come here. Yes, I need money. But you want to do a change for somebody.’ (CHW, C2)

## Discussion

The key findings indicate a definite need for palliative care in the community. Uncertainty exists over the optimal structure of a home-based palliative care service to effectively address this need, including whom to task with this service. While CHWs’ core responsibility is disease prevention and health promotion, their presence in the community alerts them to those in need of palliative care. The absence of a supporting home-based care service leaves them in a precarious position between official scope of practice and community needs.

The high prevalence reported and heavy burden of disease-related symptoms and the biopsychosocial nature of patients’ suffering are in keeping with the literature.^5,6,7^ Poor analgesic adherence and uncontrolled pain was a common finding. Patients and caregivers were uncertain about managing pain and other symptoms and did not know who to contact for help. Limited financial resources hampered families’ caregiving capacity. In addition to practical and financial support, patients and caregivers needed more information on symptom management, nutrition, basic care procedures and dealing with emotional distress. It was important for help to be available throughout the disease course. This concurs with published reviews indicating practical nursing assistance, information on symptom control and caregiving, effective communication and psychosocial and spiritual support as home-based palliative care recipients’ unmet needs.^21,22^

Home is often the core location where palliation is needed. Participants spent most of their time at home and found it challenging to attend primary healthcare clinics or the local intermediary care facility. This was because of practical and financial hindrances and personal preference. These findings correspond with published evidence where the majority of patients preferred their homes as place of end-of-life care and death.^23^ It also implies that while inpatient and clinic-based care is a central part of comprehensive palliation and may supplement domiciliary care, it does not refute the need for an effective home-based palliative care service which has been proven to lessen symptom burden and enable patients to die at home.^24^

Because the better part of palliation is needed at patients’ homes, palliative care should ideally be integrated into primary healthcare systems.^25^ According to the World Health Organization (WHO), a national palliative care policy, the inclusion of palliative care in disease-specific legislation, the availability of essential medicines (notably opioids) and adequate palliative care education are indicators of enhanced access to palliative care in primary healthcare.^25^ The development and adoption of SA’s NPFSPC was a significant step towards system integration.^9^ In addition, morphine and adjuvant analgesics are available at the primary healthcare level.^26^ All patients who partook in this study had access to morphine, with some HCBC teams ensuring that patients had an adequate supply of morphine and assisting with the delivery thereof to home-bound patients.

An additional requirement for the effective integration of palliation into primary healthcare is to embed palliative care services into healthcare systems at all levels.^25^ By exploring the palliative care roles and responsibilities of CHWs as agents of primary healthcare,^11^ this study calls attention to the level of palliative care integration at the community level. As an implementation site for a rural palliative care model, the George subdistrict has made good progress with palliative care integration at hospital and primary healthcare clinic levels.^20^ The inclusion of domiciliary palliative care services has not been established to the same extent, with a shortage of CHWs and infrequent home visits identified as challenges to effective palliative care delivery.^20^ Participants identified CHWs’ absence of palliative care responsibilities beyond patient identification and referral as a major barrier to community-based palliative care. Although this is in keeping with their scope of work as described in SA’s NPFSPC,^9^ there are no HBCs in the area to complement CHWs’ tasks as is foreseen in the policy.

Given CHWs’ limited care responsibility, home-based palliation was primarily a registered nurse’s function. Participants were concerned about the nurses’ workload and their availability to attend to home-bound patients’ palliative care needs. Since registered nurses are a scarce resource in SA, this is a valid concern.^10^ This task distribution is also different from what is recommended by leading publications. The WHO states that CHWs have a vital role in conducting regular home visits to palliative care patients.^25^ In addition to providing emotional support, CHWs can identify unmet basic and symptom-related needs.^25^ This recommendation is supported by the WHO’s guidelines on palliative care task-shifting documented in HIV and AIDS service delivery models.^27^ According to these standards, CHWs may be tasked with pain assessments, providing information on analgesic use and giving advice on nonpharmacological methods of pain control.^27^ At end-of-life, CHWs can provide practical, psychosocial and spiritual support to patients and their families.^27^

When participating CHWs did see patients with palliative care needs, they found it difficult to empower patients towards self-care rather than to provide them with basic care. While all considered information-giving an essential part of their responsibilities, CHWs had contradictory opinions on whether patients with palliative care needs and their caregivers could self-manage. Most CHWs felt that resource-poor communities’ social and financial hardships hampered their ability to care for those whose symptoms required palliation. This underscores the importance of a realistic balance between self-care and palliative care provided by the healthcare system. As stated by the WHO,^25^ while family caregivers should be equipped and supported to provide patients with the necessary basic care, co-existing responsibilities and poverty may limit their ability to do so. A related argument is the ongoing responsibility of the healthcare system to provide appropriate care to those already ill while working towards a healthier community.^28^ Participants felt that this principle was not honoured.

Community health workers who previously worked as HBCs were profoundly impacted by their reformulated job responsibilities and struggled to adjust to their new preventive and promotive roles. Communities who still expected a home-based care service added to their conflict. Similar to other reports,^14,29^ CHWs were subject to various operational barriers. They often worked in dangerous environments and were concerned about their personal safety. Physical distances and time limitations made it difficult to visit seriously ill patients in addition to doing screening and tracing visits. Daily screening quotas and the related recording and reporting process were time consuming and overwhelming. The challenges associated with CHWs’ position at the interface of communities and the formal health sector are equally well documented.^29,30^ Community health workers reported that community members held them accountable for healthcare system failures, while not all professional members of the healthcare team appeared to recognise the CHWs’ potential value. This deepened their marginalisation and led to role ambiguity, a finding confirmed elsewhere.^29,30^ Also described in the literature^17,18^ and confirmed by this study was the vicarious and often unacknowledged emotional effect patients’ suffering had on CHWs. Finally, lack of knowledge and skills presented a barrier to CHWs’ ability to contribute to palliative care delivery. The need for palliative care training, should CHWs actively participate in care delivery, is similar to that found by other studies.^17,18^

Community health workers have attributes that make them uniquely suited for home-based palliative care delivery. Being part of the communities they served, CHWs displayed a deep understanding of the socio-economic challenges patients faced and the multidimensional nature of their suffering. Provided that their other tasks allow them time to perform home visits, CHWs’ constant presence in the community can contribute to care continuity. Those who previously worked as HBCs were familiar with basic nursing tasks, elementary symptom assessment, treatment adherence and the importance of psychosocial support. This suggests that, with the necessary training, CHWs should be able to master the palliative care duties identified by the WHO as within their scope of ability.^27^ Similar to previous findings,^29^ CHWs’ displayed a high level of internal motivation and were passionate about patient care.

Despite their potential to contribute to a palliative care service, this study suggests that not all CHWs are equally suited to care for patients at the end of life. Some found it hard to cope with the associated strain, while others battled to integrate their palliative care responsibilities with their preventive and promotive tasks. These observations relate to the concept of medical generalism, an appealing option in particularly resource-poor settings as it aims to improve equity and overall health status.^31^ In SA’s primary healthcare system, CHWs are considered generalists, responsible for comprehensive care delivery (including palliation) to patients of all age groups.^32^ As reported elsewhere,^31,32^ this study confirms that care must be taken not to lose sight of CHWs’ limitations, both in terms of their inherent abilities and the resources available to them, as excessively high expectations may lead to work overload and role fragmentation and conflict.^11^

This study illustrates the impact of broader system-related influences on CHWs’ performance. While they may have the characteristics to add to community-based palliative care, CHWs’ knowledge, skills and attitudes only partly determine their contribution.^33^ It requires the alignment of an array of healthcare system and community factors to maximise their impact on health-related outcomes.^33^ To optimise CHWs’ palliative care functions, their potential roles must be seen in the context of the systems in which they operate. From a societal perspective, communities’ expectations of a home-based palliative care service must be understood. While existing literature depicts the needs of those residing in developed countries,^21,22^ little is known about the home-based care needs of South Africans and their motivation to engage with such a service. Furthermore, there is an urgent need to determine the extent to which CHWs can function as generalists. The role of HBCs as a supplementary workforce and of specialised community-based teams to support CHWs’ in palliative care delivery^34^ must be clarified. Beyond the need for innovative service structures, a re-examination of perspectives on palliative care delivery within the primary healthcare milieu is necessary. Moving from disease-specific and care-oriented services towards comprehensive health-related goals demands several mind-shifts to fully appreciate the contribution of community-based health teams.^14^ This study emphasises the need to also consider the continued demand for care and treatment at the community level by those who are already ill. By addressing their needs, not only will the well-being of the broader community be enhanced through health promotion and disease prevention, but the well-being of those at the end of life will also be improved through effective symptom palliation.

## Limitations

This was a qualitative study with the aim to obtain in-depth understanding of participants’ experience of palliative care. Consequently, the findings may lack generalisability. It was conducted in a single subdistrict in SA. In addition to the differential influence of provincial healthcare legislation, the fact that the subdistrict is a pilot site for a rural palliative care project might have influenced the findings. Also, all the participating patients had a cancer diagnosis, which does not represent the scope of SA’s disease burden and the profile of the majority of patients who may need palliative care. As only patients using morphine were included, all participants’ pain had been diagnosed and treated. Combined with the requirement that patients had to live in safe and accessible areas, the study might have excluded those most vulnerable to suffering.

## Reflexive paragraph

The principal researcher is a medical doctor in private practice who works within the context of the study setting. She has a background in palliative medicine and is experienced in the provision of home-based care. The researcher considered how her position as medical doctor might have influenced the participants and how her observations were shaped by her experience in the private health sector. While the researcher recognised the risk of bias, it was also accepted as an advantage to understand in more depth the experiences and perceptions of the study participants. Through reflection and regular discussions with the research supervisors, she remained aware of and addressed potential researcher bias.

## Conclusion

The aim of this study was to explore the roles of CHWs in palliative care in the George subdistrict of the Western Cape province, SA. Four major themes emerged, confirming the need for community-based palliative care and highlighting the influence of CHWs’ characteristics and broader system-related factors on their ability to contribute to palliative care delivery. Future research should focus on describing the home-based palliative care needs of patients in similar under-resourced settings in sub-Saharan Africa and beyond, defining the optimal structure of a home-based palliative care service within primary healthcare to optimise the role of nonprofessional workers in palliative care.
